# Supplementary material for: Comparative genomics of Pseudomonas syringae pathovar tomato reveals novel chemotaxis pathways associated with motility and plant pathogenicity
Source: PeerJ. 2016 Oct 25;4:e2570. doi: 10.7717/peerj.2570 (PMC5088630; doi:10.7717/peerj.2570)
Supplement: Supplemental Information 1 [file peerj-04-2570-s004.docx]

**Supplementary Information for:**

Comparative genomics of *Pseudomonas syringae* pathovar *tomato* reveals novel chemotaxis pathways associated with motility and plant pathogenicity

Christopher R. Clarke^1^, Byron W. Hayes^1^, Brendan J. Runde^1^, Eric Markel^2^, Bryan M. Swingle^2,3^, and Boris A. Vinatzer^1^

^1^Department of Plant Pathology, Physiology, and Weed Science. Virginia Tech. Blacksburg, VA, USA.

^2^ Emerging Pests and Pathogens Research Unit, Robert W. Holley Center for Agriculture and Health, ,United States Department of Agriculture-Agricultural Research Service, Ithaca, NY, USA.

^3^Plant Pathology and Plant-Microbe Biology Section, School of Integrative Plant Science, Cornell University, Ithaca, NY, U.S.A.

**Correspondence:** [thechrisclarke@gmail.com](mailto:thechrisclarke@gmail.com)

**Contents:**

Tables S1-S2

Figures S1-S7

Author Contributions

**Other Files included in this submission**

Supplementary videos 1-3

| **Annotation** | **Start** | **End** | **Frame** | **Length(nt)** | **Accession** |
| --- | --- | --- | --- | --- | --- |
| **che1 - based on 60k-90k ctg 127 PtoT1 genome (some accession #s are from other strains)** | | | | | |
| hypothetical | 77 | 796 | -3 | 720 | NP_790769 |
| glutothione s-trans | 1010 | 1795 | -3 | 786 | ZP_03398314 |
| hypothetical | 1789 | 2145 | +1 | 357 | ZP_03398315 |
| pilin | 2426 | 3355 | -3 | 930 | ZP_03398316 |
| PilB | 3086 | 4780 | +2 | 1695 | ZP_03398317 |
| PilC | 4783 | 6000 | +1 | 1218 | ZP_03398318 |
| PilD | 6002 | 6874 | +2 | 873 | ZP_03398319 |
| dephospho-CoA kinase | 6871 | 7494 | +1 | 624 | ZP_03398320 |
| hypothetical (DNA gyr inhib) | 7491 | 7700 | +3 | 210 | NP_790761 |
| hypothetical protein | 7967 | 8293 | -3 | 327 | ZP_03398322 |
| hypothetical | 8490 | 9119 | +3 | 630 | ZP_03398323 |
| MOSC domain prot | 9116 | 9649 | +2 | 534 | ZP_03398324 |
| NADH dehydrogenase | 9866 | 11164 | +2 | 1299 | ZP_03398325 |
| mcp | 11592 | 12806 | +3 | 1215 | ZP_03398326 |
| cheY-122 | 12816 | 13184 | +3 | 369 | ZP_03398327 |
| STAS domain prot | 13211 | 13501 | +2 | 291 | ZP_03398328 |
| cheA-678 | 13614 | 15650 | +3 | 2037 | ZP_03398329 |
| - | 15684 | 15908 | +3 | 225 | - |
| mcp | 15679 | 17334 | +1 | 1656 | ZP_03398330 |
| cheW | 17371 | 17910 | +1 | 540 | ZP_03398331 |
| cheR | 17920 | 18729 | +1 | 810 | ZP_03398332 |
| cheD | 18726 | 19232 | +3 | 507 | ZP_03398333 |
| cheB | 19222 | 20298 | +1 | 1077 | ZP_03398334 |
| hypothetical | 20332 | 21543 | -1 | 1212 | ZP_03398335 |
| hopAI1 | 21712 | 22515 | -1 | 804 | ZP_03398336 |
| hopAH1 | 22682 | 23950 | -3 | 1269 | ZP_03398337 |
| hopAG1 | 24054 | 26159 | -2 | 2106 | ZP_03398338 |
| hypothetical | 26159 | 26410 | -3 | 252 | ZP_03398339 |
| sensor hist kinase | 27216 | 28829 | -2 | 1614 | ZP_03398341 |
| **che2 - Based on ctg57 155K-220K PtoT1 genome (some accession #s are from other strains)** | | | | | |
| transcriptional activ anr | 12957 | 13691 | +3 | 735 | ZP_03395676 |
| adenine phosribotransf | 13753 | 14301 |  | 548 | ZP_03395677 |
| hypothetical | 14432 | 14776 | -2 | 345 | ZP_03395678 |
| cheW | 14834 | 15313 | -2 | 480 | ZP_03395679 |
| cheW | 15371 | 16258 | -2 | 888 | ZP_03395680 |
| parA | 16334 | 17122 | -2 | 789 | ZP_03395681 |
| chemotB | 17168 | 18055 | -2 | 888 | ZP_03395682 |
| chemotA | 18062 | 18802 | -2 | 741 | NP_791807 |
| cheB | 18802 | 19974 | -3 | 1173 | ZP_03395684 |
| cheA | 20025 | 22265 | -1 | 2241 | ZP_03395685 |
| cheZ | 22299 | 23084 | -1 | 786 | ZP_03395686 |
| cheY | 23104 | 23478 | -3 | 375 | ZP_07231294 |
| flg biosynth sigma | 23732 | 24472 | -2 | 741 | ZP_03395688 |
| fleN | 24469 | 25302 | -3 | 834 | NP_791801 |
| flhF | 25418 | 26746 | -2 | 1329 | NP_791800 |
| flhA | 26758 | 28797 | -3 | 2040 | NP_791799 |
| flhB | 29272 | 30408 | -3 | 1137 | NP_791798 |
| fliR | 30410 | 31186 | -2 | 777 | NP_791797 |
| fliQ | 31202 | 31471 | -2 | 270 | NP_791796 |
| fliP | 31497 | 32249 | -1 | 753 | NP_791795 |
| flliO | 32249 | 32689 | -2 | 441 | NP_791794 |
| fliN | 32690 | 33148 | -2 | 459 | NP_791793 |
| fliM | 33210 | 34178 | -1 | 969 | NP_791792 |
| fliL | 34188 | 34691 | -1 | 504 | NP_791791 |
| fliK | 35246 | 36670 | -2 | 1425 | ZP_03395701 |
| HPT dom. Cont. | 36769 | 37077 | -3 | 309 | NP_791788 |
| response regulator | 37159 | 38724 | -3 | 1566 | ZP_03395703 |
| STAS | 38870 | 39469 | -2 | 321 | NP_791786 |
| fliJ | 39255 | 39704 | -1 | 450 | NP_791785 |
| fliI | 39711 | 41123 | -1 | 1413 | NP_791784 |
| fliH | 41059 | 41877 | -3 | 819 | ZP_03395707 |
| fliG | 41893 | 42909 | -3 | 1017 | ZP_05638959 |
| fliF | 42902 | 44686 | -2 | 1785 | NP_791781 |
| fliE | 44702 | 45031 | -2 | 330 | ZP_03395710 |
| sigma54-dep tran reg fleR | 45273 | 46688 | -1 | 1416 | ZP_03395711 |
| hypothetical | 46688 | 47902 | -2 | 1215 | ZP_03395712 |
| trans reg fleQ | 48025 | 49500 | -3 | 1476 | ZP_03395713 |
| fliS | 49999 | 50388 | -3 | 390 | NP_791775 |
| fliD | 50514 | 51986 | -1 | 1473 | ZP_03395716 |
| flaG | 52070 | 52468 | -2 | 399 | ZP_03395717 |
| fliC | 52535 | 53383 | -2 | 849 | ZP_03395718 |
| 3-oxoacyl synthase | 53713 | 54642 | -3 | 930 | ZP_03395719 |
| glycosyl transferase | 54925 | 57831 | -3 | 2907 | ZP_03395720 |
| glycosyl transferase | 57963 | 61079 | -1 | 3117 | ZP_03395721 |
| flgL | 61627 | 63219 | -3 | 1593 | ZP_03395722 |
| flgK | 63233 | 64942 | -2 | 1710 | NP_791767 |

**Table S1.** Genes in the *che1* and *che2* clusters and associated accession numbers.

| **Gene** | **Purpose** | **Forward Primer** | **Reverse Primer** |
| --- | --- | --- | --- |
| *cheA1* | Disruption | AAAAAGGGCCCTGATTgcaaagccctgtggttgc | AAAAAATCGATTCATatgacccttggccggttt |
| *cheA2* | Disruption | AAAAAGGGCCCTGATgctagcgcctctacgaggtt | AAAAAATCGATTCATTacgaattcgaaaacctgctg |
| *cheA1* | Disruption - integration check | accggcactgacctcgtt | cgatttcaatgacgatgctg |
| *cheA2* | Disruption - integration check | gcgtttcgataccgtggtc | ctcgtttgacagtgctgtgg |
| *cheA1* | Cloning | AAAAACTCGAGGAAGCCCGTGAGCTGTTG | AAAAAGAGCTCCAAATCCGTCAGCGAGAAG |
| *cheA2* | Cloning | AAAAACTCGAGTTAGGGAGCACCCCATGA | AAAAAGAGCTCGCAACCCCGGATTCAAATA |
| *cheA1* | Deletion | GGGTGTCGTGCTGTAACGCGTCTATTTGAAGTACCGCCGGGTGATCGACAAAGGAAGTCATCGTGAGCATTAATCTCGATGTGGAGGCTGGAGCTGCTTC | TGGTGGAAATCTTCAGATCGTAGAACCATTTCATCGGGTTACTCCTTGGAATTGCAAATCCGTCAGCGAGAAGCGGGCGACATATGAATATCCTCCTTAG |
| *cheA1* | Sequencing | CCCTGGAGGTCAGCATGTCTACAA |  |
| *cheA1* | Sequencing | CTCAGCAGCTTCCTGCTCCATCT |  |
| *cheA1* | Integration check and sequencing | CCGAAGCAGCTCGCATGGA | TGTCGATCAGGATGATCTGG |
| *cheA1* | Sequencing | GGTCATCCAGCGCGAGGC |  |
| *cheA1* | Sequencing | CTGAGCGGCCTGATTCACCT |  |
| *cheA1* | Sequencing | GTTTGCTGCCGCAGATCGC |  |
| *cheA1* | Sequencing | CTGACGATCTACACCGTGACCGAA |  |
| *cheA1* | Sequencing | CCCTGGAGGTCAGCATGTCTACAA |  |
| *cheA1* | Sequencing | CTCAGCAGCTTCCTGCTCCATCT |  |
| *cheA1* | Sequencing | GCTGAACATCTGACGATCCTGGT |  |
| scar | Deletion scar check | GTGTAGGCTGGAGCTGCTTC |  |
| scar | Deletion scar check | CATATGAATATCCTCCTTA |  |
| *cheA2* | *cheA-2* deletion 5' Flank | CGAGGAAAGCACGCCGATCA | GAAGCAGCTCCAGCCTCCACGGCGCCGAAGCTCATGGG |
| *cheA2* | *cheA-2* deletion 3' Flank | CTAAGGAGGATATTCATATGGCCGCTCGGCGTATTTGA | GCAGGCGCACGACTGGACA |
| *cheA2* | Integration check and sequencing | ATCAGAGCAGCCGATTGTCTG | CTGGTGACCGAAGTAGAGGGCA |
| *cheA2* | Sequencing | GGCCGAAGAGGTGTCGCA |  |
| *cheA2* | Sequencing | GGCAGTGCCGGTGGTGGT |  |
| *cheA2* | Sequencing | CGTCTTTCCTACGTCGTCAGGCT |  |
| *cheA2* | Sequencing | CAGGCGTGTGGTGAGCATGA |  |
| *cheA2* | Sequencing | GACAAGGGTGAAGGTCCGCA |  |
| *cheA2* | Sequencing | CTTGCCGTTGGTGGCGGT |  |
| *cheA2* | Sequencing | CGTCTTTCCTACGTCGTCAGGCT |  |
| *cheA2* | Sequencing | CAGGCGTGTGGTGAGCATGA |  |
| *cheA2* | Sequencing | CGTCCAGAGCATCCAGCGT |  |

**Table S2.** Primers used in the study.

**
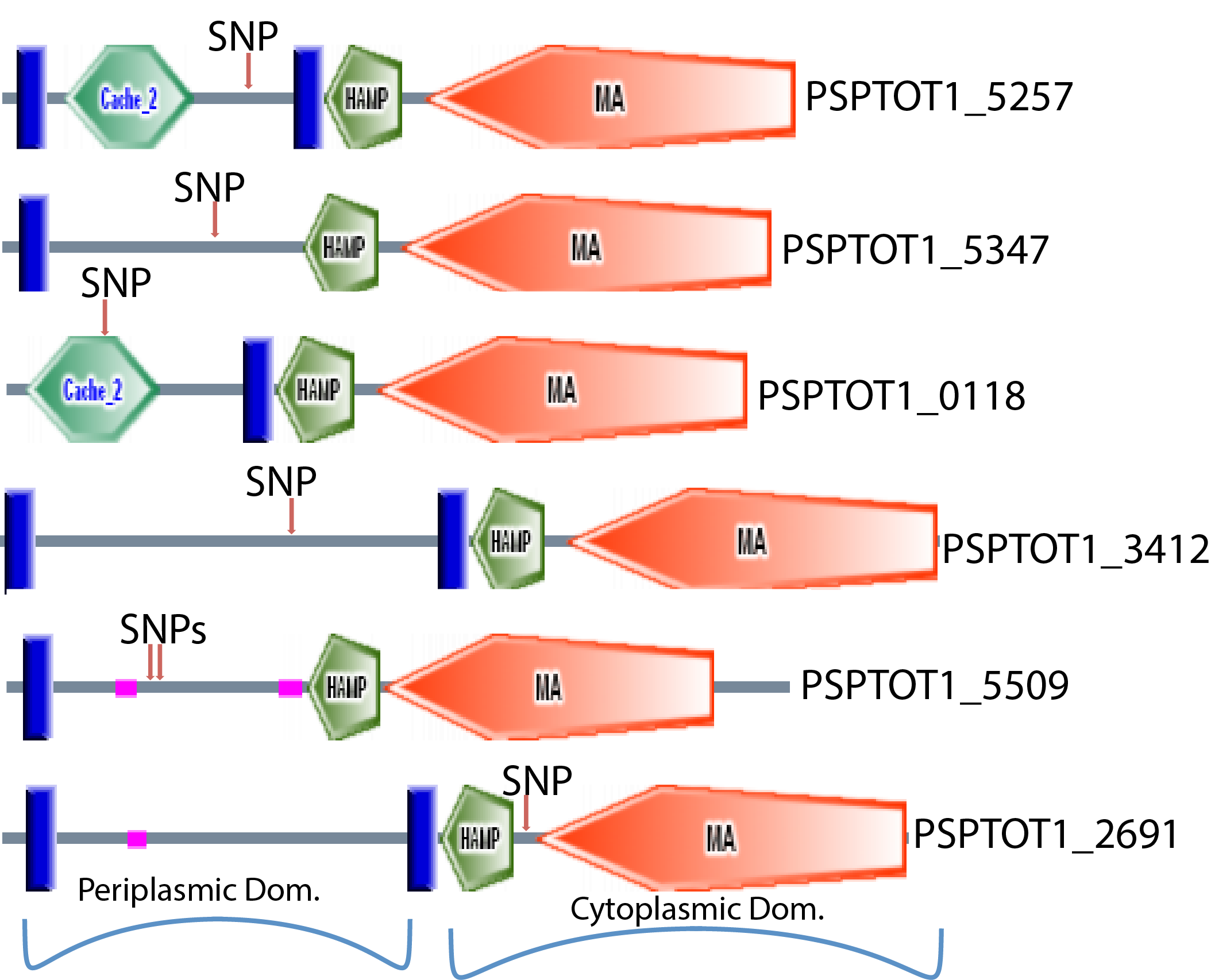
**

**Figure S1.** Location of single nucleotide polymorphisms in methyl-accepting chemotaxis proteins


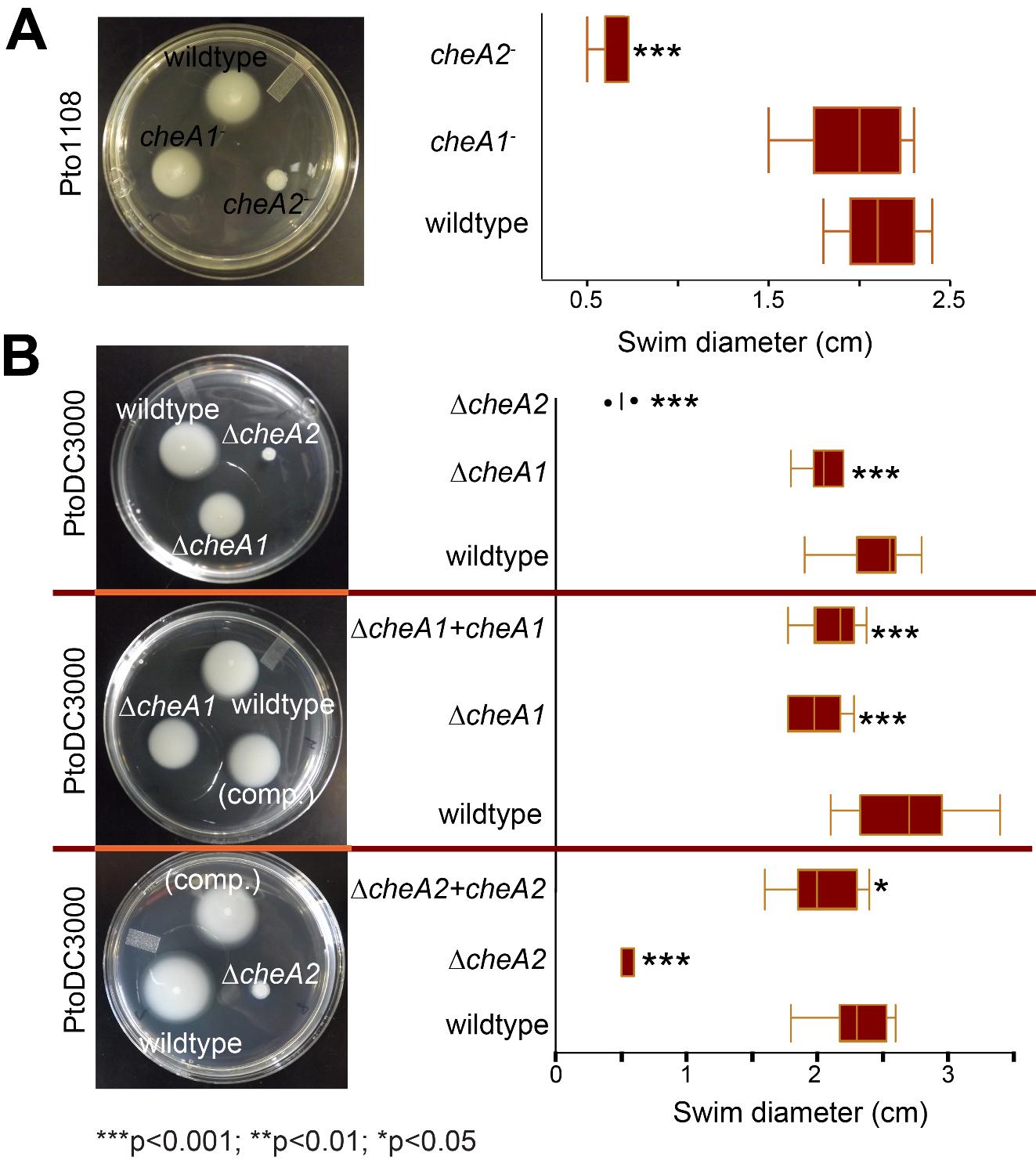


**Figure S2.** Example pictures and box plots of the colony diameter two days after inoculation of Pto1108 chemotaxis disruption strains (**A**) or PtoDC3000 chemotaxis deletion strains (**B)** on 0.28% agar KB swim plates. n = 10 and essentially identical results were obtained in 3 independent experiments. * indicates statistical difference between mutant strain and wildtype and the indicated p-value using a Student’s *t-test*.

**
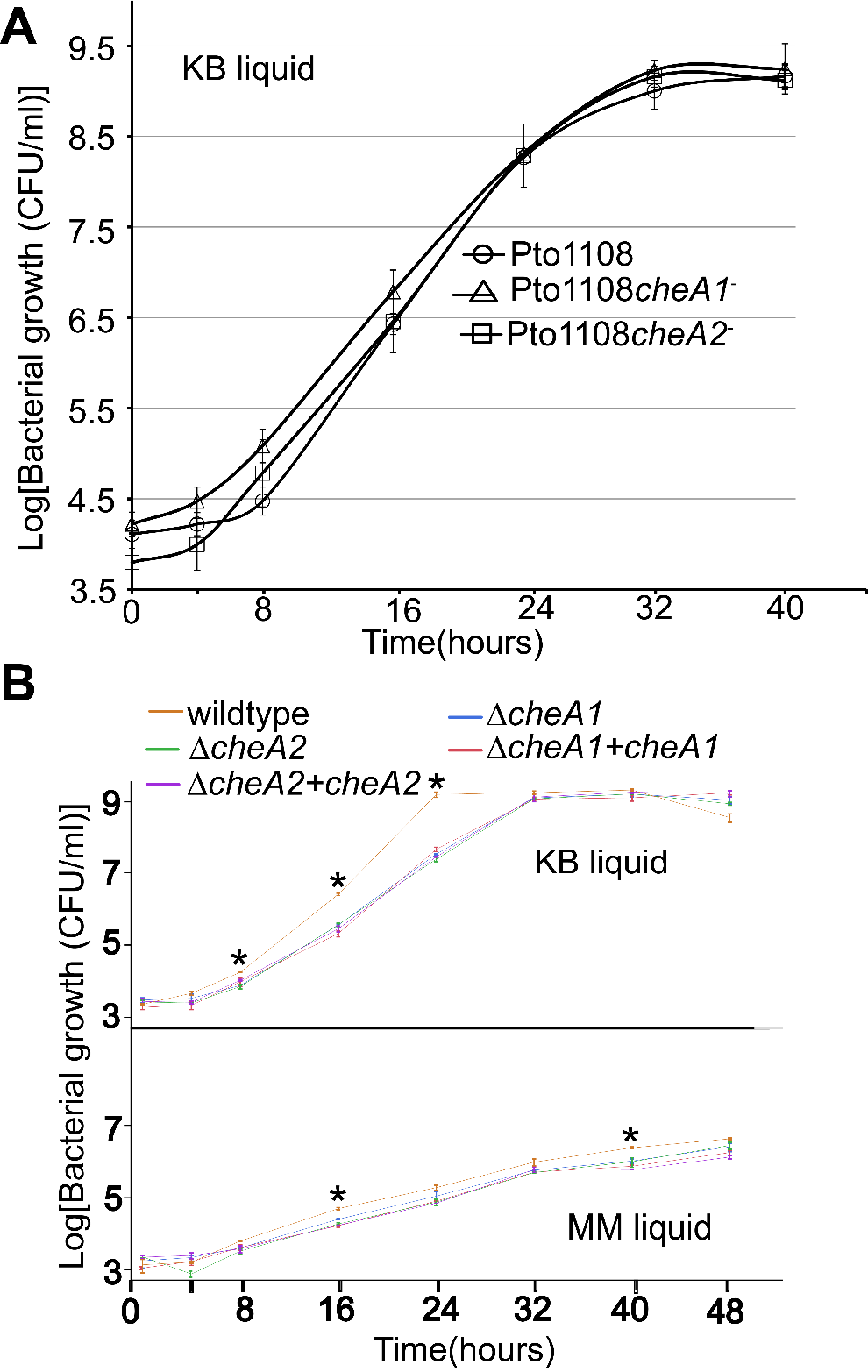
**

**Figure S3.** Growth of Pto1108 chemotaxis disruption mutants (**A**) and PtoDC3000 deletion mutants (**B**) in liquid culture. For A, n= 8. For B, n=3 and essentially identical results were obtained in 2 independent experiments. * indicates that the wildtype strain had a significantly different (p<0.01) population density than all other strains at that time point.

**
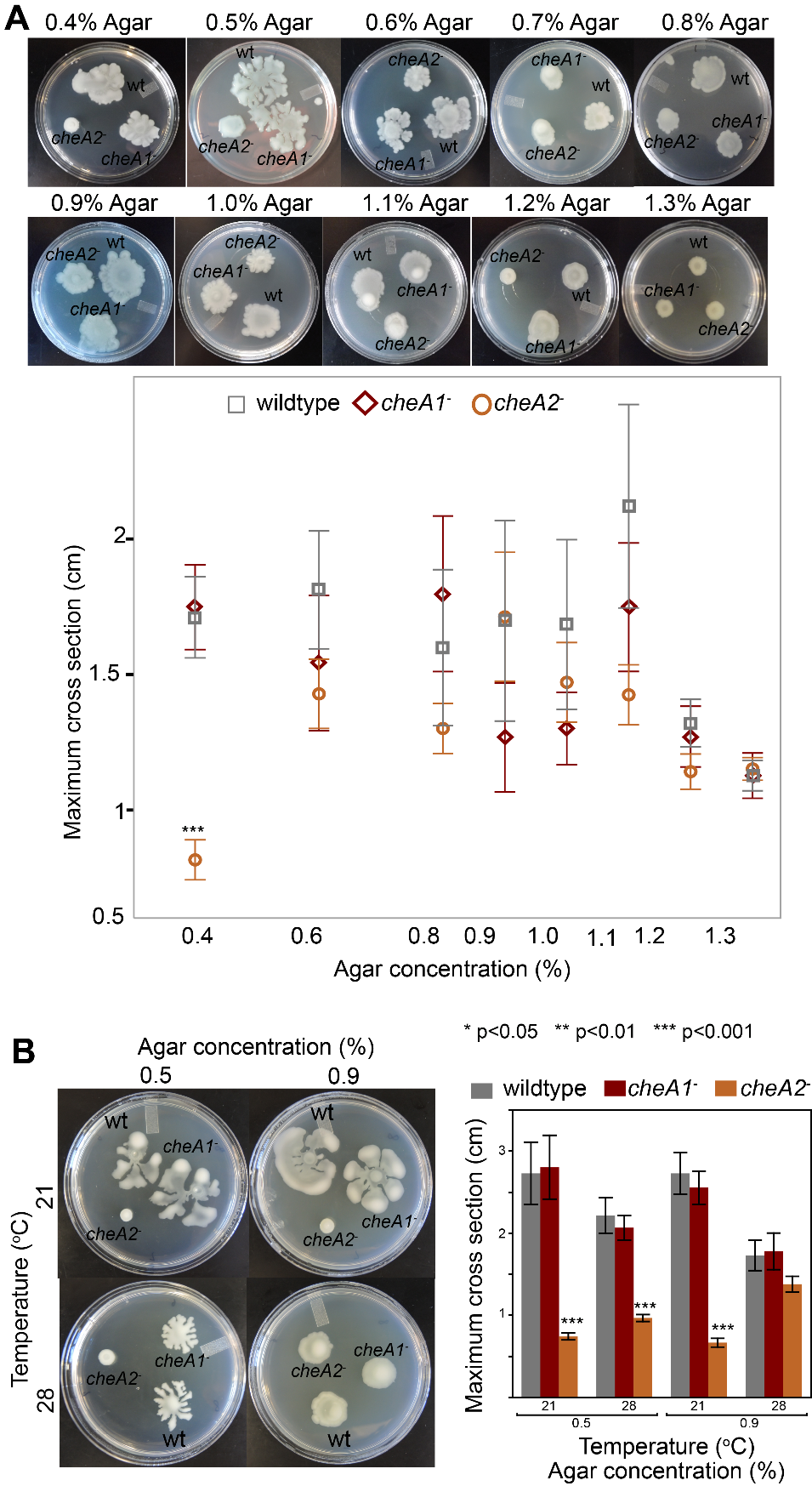
**

**Figure S4.** Example pictures and plotted average maximum swarm diameter of strain Pto1108 chemotaxis disruption mutants at 21^o^C (**A**) or 28^o^C or 21^o^C (**B**). n=8. * indicates significant differences in swim diameter for any strain between the two temperatures at the indicated p-values using a Student’s *t-test*.

**
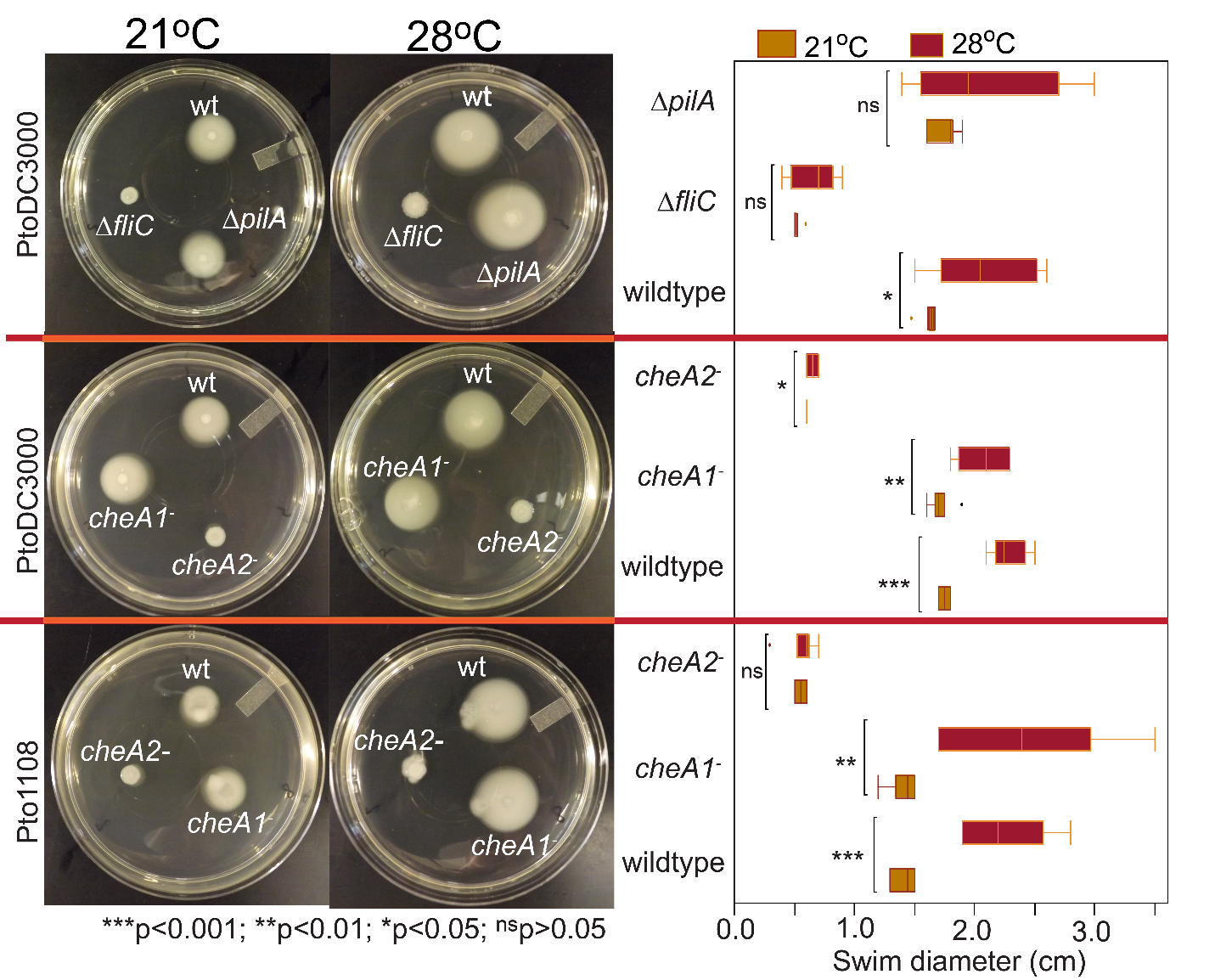
**

**Figure S5.** Example pictures and box plots of the colony diameter two days after inoculation of the indicated strains on 0.28% agar KB swim plates at 28^o^C or 21^o^C. * indicates significant differences in swim diameter for any strain between the two temperatures at the indicated p-values using a Student’s *t-test*. The smaller swim diameter is reflective of the slower growth rate of Pto at 21^o^C. No mutant strain has altered swimming motility relative to wild type due to temperature.

**
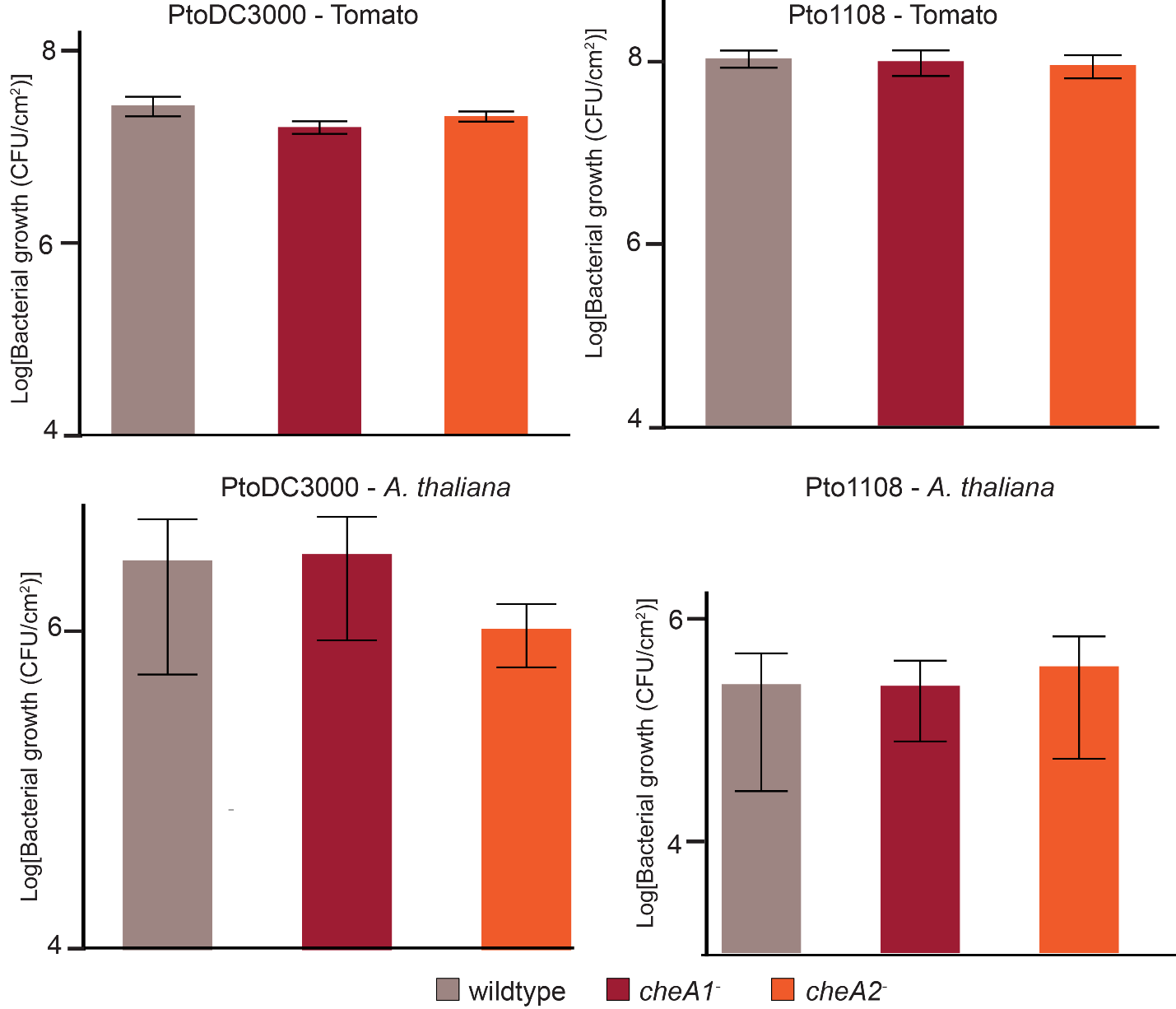
**

Figure S6. Growth of PtoDC3000 or Pto1108 on tomato (*S. lycopersicum* cv. Rio Grande) or *A. thaliana* ecotype Columbia 4 days following syringe infiltration inoculation at 0.001 (*A. thaliana*) or 0.0001 OD_600_ (tomato). Data represent the average of 4 replicates and error bars are the standard error. Similar results were obtained in at least 4 independent experiments for all sections.

**
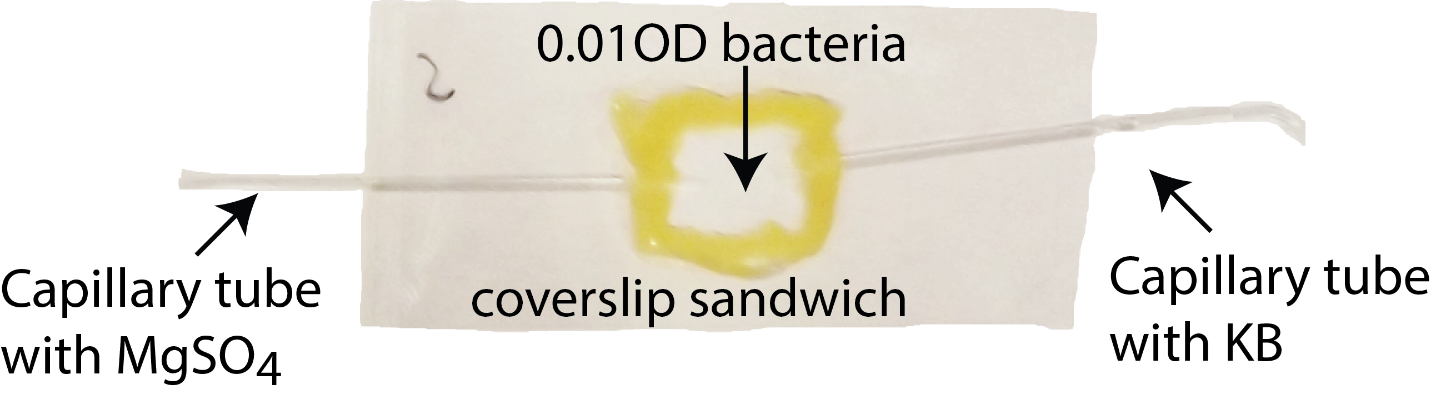
**

**Figure S7.** Setup for chemotaxis split capillary assay.

**Author contributions:** CRC and BAV designed the research; CRC performed the experiments with assistance from BWH and BJR; EM and BMS created the *cheA* deletion strains; CRC wrote the manuscript with subsequent editing from the other co-authors.
